# Role of molecular testing in the multidisciplinary diagnostic approach of ichthyosis

**DOI:** 10.1186/s13023-016-0384-4

**Published:** 2016-01-13

**Authors:** Andrea Diociaiuti, May El Hachem, Elisa Pisaneschi, Simona Giancristoforo, Silvia Genovese, Pietro Sirleto, Renata Boldrini, Adriano Angioni

**Affiliations:** Dermatology Unit, Bambino Gesù Children’s Hospital, IRCCS, Piazza S. Onofrio 4, 00165 Rome, Italy; Molecular Genetics Laboratory, Medical Genetics Unit, Bambino Gesù Children’s Hospital, IRCCS, Rome, Italy; Department of Pathology, Bambino Gesù Children’s Hospital, IRCCS, Rome, Italy

**Keywords:** Ichthyosis, Multidisciplinary approach, Molecular diagnosis

## Abstract

**Background:**

The term ichthyosis describes a generalized disorder of cornification characterized by scaling and/or hyperkeratosis of different skin regions. Mutations in a broad group of genes related to keratinocyte differentiation and epidermal barrier function have been demonstrated to play a causative role in disease development. Ichthyosis may be classified in syndromic or non-syndromic forms based on the occurrence or absence of extracutaneous signs. In this setting, the diagnosis of ichthyosis is an integrated multistep process requiring a multidisciplinary approach in order to formulate the appropriate diagnostic hypothesis and to address the genetic testing.

**Methods:**

Due to the complex features of the different ichthyoses and the high number of genes involved we have investigated a group of 64 patients, affected by syndromic and non-syndromic diseases, using Next Generation Sequencing as a new tool for the molecular diagnosis.

**Results:**

Using this innovative molecular approach we were able to find pathogenic mutations in 53 out of 64 patients resulting in 82.8 % total detection rate. An interesting result from the analysis of the data is the high rate of novel sequence variations found compared to known mutations and the relevant rate of homozygous mutations.

**Conclusions:**

The possibility to analyze a large number of genes associated with various diseases allows to study cases with phenotypes not well-determined, giving the opportunity to make new genotype-phenotype correlation. In some cases there were discrepancies between clinical features and histology or electron microscopy and only molecular analysis allowed to definitively resolve the diagnostic dilemma. The genetic diagnosis of ichthyosis leads to a more accurate and effective genetic counseling, allowing correct evaluation of the risk of recurrence, particularly in families with consanguineous background.

**Electronic supplementary material:**

The online version of this article (doi:10.1186/s13023-016-0384-4) contains supplementary material, which is available to authorized users.

## Background

The ichthyoses are a heterogeneous group of inherited skin disorders characterized by a defect of keratinization with the clinical appearance of scaling or hyperkeratosis or both. The classification of Oji et al. in 2010 has divided the various forms of ichthyosis in two major groups, non-syndromic and syndromic, on the basis of the absence or presence of extracutaneous symptoms [1]. The latter are then classified according to the prevalent symptom. Specifically, there are forms associated with neurological involvement, hair abnormalities and, in some cases, with fatal course. Apart, there are syndromic X-linked ichthyoses and those forms that do not fit in previous groups.

Non syndromic forms are divided in common ichthyoses (vulgaris and X-linked), autosomal recessive congenital ichthyosis (ARCI), keratinopathic ichthyoses and others.

ARCI is a heterogeneous group with a limited correlation between genotype and phenotype, which ranges from harlequin ichthyosis to lamellar ichthyosis (LI) and congenital ichthyosiform erythroderma (CEI). Mutations in *TGM1* are responsible for 32 % of ARCI and are strongly associated with collodion baby [2]. In this group truncating mutations cause a more severe phenotype compared to missense mutations [3].

The genetic basis of most ichthyosis forms has been unraveled over the past two decades. Nevertheless, in some patients affected by a clinically well-defined subtype of ichthyosis, the pathogenetic mutations cannot be identified (22 % in ARCI) [2].

The classification based on clinical features elaborated by the multicenter consensus [1] has been extremely useful in order to provide a correct diagnostic approach, to guarantee an early diagnosis and to reduce the medical costs. Ichthyosis diagnosis requires a multidisciplinary approach, coordinated by a dermatologist, in order to correlate the clinical features, the microscopic and laboratory data and to formulate a diagnostic hypothesis; the final diagnosis is generally established through molecular testing.

Currently, molecular diagnosis of congenital and syndromic ichthyoses is a very hard challenge due to the high number of genes involved. Moreover, there is a considerable genetic heterogeneity, since multiple genes can cause the same form of ichthyosis, and phenotypic heterogeneity, since the same gene may cause different forms of ichthyosis.

Sanger sequencing has been previously recognized as the gold standard for mutation analysis in molecular diagnosis; however, its low-throughput, long turnaround time and overall cost [4] have called for new paradigms. Next-Generation Sequencing (NGS) can massively sequence millions of DNA segments, promising low costs, increased workflow speed and enhanced sensitivity in mutation detection [4, 5]. Based on these observations we have planned to study a group of ichthyosis patients by means of NGS using a large panel of genes able to cover the mutational spectrum of most disease forms.

Our goal is to validate a multidisciplinary approach and to improve diagnostic accuracy in patients with ichthyosis achieving the identification of the mutation responsible for the disease.

## Methods

### Patient recruitment

In this study we enrolled, from September 2013 until December 2014, 64 patients, 34 males and 30 females (age ranges from few months to 66 years), affected with ichthyosis from the Pediatric Dermatology Unit and from the Molecular Genetics Laboratory, Medical Genetics Unit Outpatients Department of the Bambino Gesù Children’s Hospital in Rome;. The study was approved by Ethical committee scientific board of Bambino Gesù Children’s Hospital and was conducted in accordance with the Helsinki Declaration. Patients were referred to four distinct groups: ARCI, X-linked ichthyosis, keratinopathic ichthyosis and syndromic forms while individuals with ichthyosis vulgaris, which is not a rare disease, were excluded from the study. After obtaining informed consent, blood samples were drawn from patients for mutation search and from parents for mutation validation and segregation analysis. Genomic DNA was extracted from peripheral blood with Qiagen columns (QIAamp DNA minikit; Qiagen, Hilden, Germany) according to the manufacturer’s instructions. Concentration and purity of DNA samples were quantified by ND-1000 spectrophotometer (NanoDrop; Thermo Scientific, Waltham, MA, USA).

### Clinico-pathological approach

Our diagnostic path first provides gathering a thorough history specifically oriented to family history, pregnancy, birth, and presentation of the disease. Secondly, dermatological examination must consider the type (size, thickness, colour) and distribution of scales, the presence of erosions, the involvement of palms and soles.

In all cases, according to the history and to the clinical appearance, we investigate possible extracutaneous symptoms, together with other specialists when required (pediatrician, neurologist, ophthalmologist, specialist in metabolic disorders, and otolaryngologist).

In case of a clear clinical diagnosis of ichthyosis vulgaris we do not proceed to further study, while all other patients undergo skin biopsy. In particular we perform biopsy even in those cases that, while presenting a phenotype compatible with the vulgaris type, show inconsistent anamnesis or suspicion of extracutaneous involvement.

Skin sample is subjected to light and electron microscopy and, in selected cases, immunohistochemical examination according to the suspected diagnosis. Microscopic hair examination is performed in case of suspicion of Netherton syndrome or trichothiodystrophy.

When extracutaneous involvement is detected, the organ specialists are involved in a multidisciplinary approach in order to orientate specific laboratory tests (fitanic acid, very-long-chain-fatty-acid, liver function tests, creatine phosphokinase etc.) or other instrumental examinations. Finally, the case is presented to the geneticist with a diagnosis based on clinicopathological findings.

In case of blood sample sent directly to our Molecular Genetics Laboratory we ask for clinic-pathologic information, pictures of the patient, family history and laboratory data. All this information is collegially discussed by the dermatologist, geneticist and pathologist.

### Genetic investigations: Targeted re-sequencing

Targeted resequencing was performed with a uniquely customized design: TruSeq® Custom Amplicon (Illumina, San Diego, CA) using the MiSeq® sequencing platform (Illumina). TruSeq Custom Amplicon (TSCA) is a fully integrated end-to-end amplicon sequencing solution, including online probe design and ordering through the Illumina website, assay, sequencing, automated data analysis and offline software for reviewing results. Online probe design was performed by entering into the Design Studio (DS) software (Illumina) the target genomic regions. DS is a personalized, easy-to-use, web-based sequencing assay design tool that enables to move from project initiation to design, review, and ordering. DS provides dynamic feedback to optimize target region coverage, reducing the time required to design custom projects. Once the design is completed, a list of amplicons (short regions of DNA covering the full target region) is visualized and their quality is assessed on the basis of the predicted amplicon score provided by DS. The amplicon score is an estimate of the relative performance of a particular amplicon compared to all others in the pool. DS returns only candidate amplicons that are predicted to work well in the multiplex TruSeq Custom Amplicon assay. TSCA kit produces the required targeted amplicons with the necessary adapters and indices for sequencing on the MiSeq® system without any additional processing. Library preparation and sequencing runs have been performed according to the manufacturer’s procedure.

A TSCA panel design has been generated to investigate the genes involved in syndromic and non-syndromic ichthyoses: all coding regions, exon-intron boundaries, 5’UTR and 3’UTR of all the genes. A panel of 1080 amplicons, each of 425 bp was designed, with a total length of 266,754 bp. The total coverage obtained by DS across the entire region of interest was 95 % with amplicons showing scores in the range of 60 % – 100 %. Amplicons with a score lower than 60 % were excluded from the TSCA panel. The list of genes included in the panel is reported in Additional file 1: Table S1. Genes associated with non-syndromic ichthyosis and those associated with syndromic ichthyosis were merged into a single panel to facilitate laboratory procedures.

### Data analysis

The MiSeq® system provides fully integrated on-instrument data-analysis software. The MiSeq Reporter software performs secondary analysis on the base calls and quality scores generated by Real Time Analysis (RTA) during the sequencing run. The type of analysis performed is based on the analysis workflow selected. The TruSeq Amplicon workflow evaluates short regions of amplified DNA, or amplicons, for variants. Focused sequencing of amplicons enables high coverage of particular regions across a large number of samples. The TruSeq Amplicon workflow performs demultiplexing of indexed reads, generates FASTQ files, aligns reads to a reference, identifies variants, and writes output files to the Alignment folder. SNPs and short indels are identified using the Genome Analysis Toolkit (GATK). GATK calls raw variants for each sample, analyzes variants against known variants, and then calculates a false discovery rate for each variant. Each single variant has been evaluated for the coverage and the Qscore, and visualized *via* Amplicon Viewer (AV) and Integrative Genome Viewer (IGV) software [6, 7]. The Qscore is the prediction of the probability of an erroneous base call, in particular, a value of Q30 represents the probability to call an erroneous base out of 1000, reflecting an accuracy of the sequenced base of 99.9 %. All detected variants have been filtered based on their Qscore: only variants showing Qscore > 30 have been considered in this study. Coverage for a defined amplicon is the average number of sequencing reads representing a given nucleotide in that amplicon. All mutations identified by Miseq Reporter were validated by Sanger sequencing or array Comparative Genomic Hybridization (aCGH) in cases of genomic rearrangements, using standard protocols.

## Results

Sequencing data generated were evaluated on the basis of the Qscore and coverage. As predicted by DS coverage indication, the analysis confirmed the coverage of 95 % for the panel. To achieve a full coverage of the whole target regions, these ones were completed by conventional Sanger sequencing.

As regards non-syndromic ichthyosis gene analysis we have sequenced twenty regions by Sanger. For the syndromic genes: *ALDH3A2* and *MBTPS2* were fully covered by NGS analysis; *PHYH*, *PEX7*, *SNAP29*, *AP1S1*, *SPINK5* and *EBP* had only one region to be sequenced by Sanger, while *VPS33B* six regions.

Each group of patients was investigated on the basis of the clinicians and the pathologist information with a specific diagnostic indication. Whenever discordance with the genetic results was present, the case was carefully reconsidered in order to reach a correct diagnosis.

Tables 1, 2, 3 and 4 summarize personal data, family history, clinical findings and histopathological/ultrastructural diagnosis of our population.

**Table 1:** Clinicopathological findings in autosomal recessive congenital ichthyosis (ARCI) patient group

Target gene	Sample ID	Disease onset	Sex	Family history	Age at examination	Dermatological features	Extracutaneous findings	Histopathology/Immunopathology/Electron microscopy^c^
NIPAL4	ID-1	1 year	F	No	5 yrs	Brown scales, PP (+)	-	ARCI
	ID-2	Birth	M	Yes^a^	2 yrs	Scales	-	NA
	ID-3	Birth	M	No	22 yrs	Large brown scales, hyperkeratotic plaques	-	ARCI
	ID-4	Birth	M	No	24 yrs	Face erythema, Hyperkeratotic plaques , PP (+++), ectropion,	-	Keratinopathic ichthyosis
ALOX12B	ID-5	Birth	M	No	2 yrs	Face erythema, lamellar scales, PP (+), ectropion	-	ARCI
	ID-6	Birth, collodion	M	No	13 yrs	Face erythema, large scales, PP (++)	-	ARCI
	ID-7	Birth	M	No	5 yrs	Erythema, thin and large scales, PP (+)	-	ARCI
	ID-8	NA		NA		NA	NA	NA
	ID-9	NA	M	No	72 yrs	Generalized erythema, large scales	-	ARCI
	ID-10	Birth, collodion	F	No	1 mo	Fine scales, minimal erythema, PP (+), ectropion, ear deformity	-	ARCI
	ID-11	NA	F	No	6 yrs	Large and medium brownish scales	-	NA
	ID-12	Birth, collodion		No	Birth	Whitish and thin scales, PP (++), ectropion, eclabion	-	ARCI
	ID-13	Birth, collodion	M	No	9 yrs	Fine whitish scales, particularly on the abdomen	-	Undefined
CYP4F22	ID-14	NA	NA	NA	NA	NA	NA	NA
	ID-15	Birth, collodion	F	No	1 yrs	Rapid detachment of collodion and almost complete normalization of the skin (fine scaling), PP (+)	-	NA
TGM1	ID-16	NA	NA	NA	NA	NA	NA	NA
	ID-17	Birth, collodion	M	No	16 yrs	Large brownish scales, erythema on face, PP (++), ectropion	-	ARCI
	ID-18	Birth, collodion	F	No	3 yrs	Small whitish scales, PP (+), ectropion	-	ARCI
	ID-19	Birth, collodion	F	No	4 mos	Scales	-	NA
	ID-20	Birth, collodion	F	No	7 mos	Large brownish scales, onychodystrophy, PP (++), ectropion, eclabion, ear deformity	-	ARCI
	ID-21	Birth, collodion	M	No^b^	1 yr	Brownish large scales, slight erythema, PP (++), ectropion, eclabion	Prematurity, cryptorchidism	ARCI
	ID-22	Birth	F	No	1 yr	Large scales	Psychomotor delay, pancytopenia,hepatomegaly	NA
ALOXE3	ID-23	Birth	M	No	1 yr	Fine small scales	-	ARCI
	ID-24	Birth, collodion	M	No	3 yrs	Small thin scales, PP (++), ectropion, eclabion, ear deformity	-	ARCI
ABCA12	ID-25	Birth	M	No^b^	15 yrs	Harlequin ichthyosis	NA	NA
	ID-26	NA	F	No	6 yrs	NA	NA	A
	ID-27	NA	F	NA	36 yrs	NA	NA	NA
**/**	ID-28	3 months	F	No	5 yrs	Small whitish scales	-	ARCI
**/**	ID-29	Birth	M	No	38 yrs	Erythema, brownish scales, onychodystrophy, PP (++)	-	NA
**/**	ID-30	Birth	F	No	26 yrs	Large scales	-	NA
**/**	ID-31	Birth	F	NA^d^	1 yr	Erythroderma, large whitish scales, hypotrichosis, onychodystrophy, PP (++), ectropion, ear deformity	Mental delay, cleft palate	Undefined

^a^Sister, ^b^Consanguineous parents; *NA* not available, *Yr(s)* year(s), *mo(s)* month(s), *PP* palmoplantar involvement; (+) hyperlinearity; (++) mild/moderate keratoderma; (+++) pronounced keratoderma. ^c^Diagnosis based on histopathological/immunohistochemical/ultrastructural findings; ^d^the patient was adopted

**Table 2:** Clinicopathological findings of X-linked ichthyosis patient group

Target gene	Sample ID	Disease onset	Sex	Family History	Age at examination	Dermatological features	Extracutaneous findings	Histopathology/Immunopathology/Electron microscopy^d^
*STS*	ID-47	NA	M	Yes^a^	17 yrs	Brown scales	-	NA
	ID-48	NA	M	Yes^a^	16 yrs	Brown scales	-	NA
	ID-49	1 yr	M	Yes^b^	8 yrs	Brownish polygonal scales, sparing of folds	-	ARCI
	ID-50	NA	M	NA	3 yrs	NA	NA	NA
	ID-51	NA	M	NA	10 yrs	NA	NA	NA
	ID-52	Birth, collodion	M	Yes^a^	5 mos	Fine scales on the trunk and brownish on the arms and legs, residue of collodion membrane, PP (+)	Failure to thrive	Undefined
	ID-53	Birth	M	No	16 yrs	Brownish scales on legs and arms, large scales on the trunk, sparing of folds, involvement of the scalp	-	ARCI
	ID-54	1 yr	M	No	18 mos	Small and medium brownish scales, sparing of folds	-	X-linked
	ID-55	3wks	M	Yes^c^	12 mos	Medium scales and onychodystrophy, PP (+)	-	X-linked
	ID-56	NA	M	NA	12 yrs	Brown scales	Learning disabilities	NA
	ID-57	Birth	M	No	1yr	Brown scales	Cystine calculi, behavioural anomalies	NA
	ID-58	Birth	M	No	6 yrs	Small and medium brown scales, sparing of antecubital and popliteal areas	Hyperactivity	Ichthyosis vulgaris
	ID-59	NA	M	No	3 yrs	Brownish scales	Autistic behaviour and language delay	NA

^a^Brother; ^b^Maternal grandfather and two maternal uncles; ^c^Maternal uncle; NA: not available; Yr(s): year(s); mo(s): month(s); (wks) weeks; PP: palmoplantar involvement; (+) hyperlinearity; (++) mild/moderate keratoderma; (+++) pronounced keratoderma. ^d^Diagnosis based on histopathological/immunohistochemical/ultrastructural findings

**Table 3:** Clinicopathological findings in keratinopathic ichthyosis patient group

Target Gene	Sample ID	Disease onset	Sex	Family history	Age at examination	Dermatological features	Extracutaneous findings	Histopathology/Immunopathology/Electron microscopy^c^
KRT1	ID-60	1 yr	M	Yes^a^	49 yrs	Hyperkeratosis, PP (++)	Bilateral syndactyly of the II/III toes	NA
KRT10	ID-61	Birth	F	NA	1 yr	NA	NA	NA
	ID-62	NA	M	NA	2 yrs	Cutaneous erosions, hyperkeratosis over the joints, PP (++)	-	Keratinopathic ichthyosis
KRT2	ID-63	Birth	F	Yes^b^	27 yrs	Diffuse hyperkeratosis, particularly over the joints, few erosions, PP (+)	-	Keratinopathic ichthyosis
	ID-64	NA	M	NA	36yrs	NA	NA	NA

^a^Mother and grandmother; ^b^daughter; *NA* not available, *Yr(s)* year(s), *mo(s)* month(s), *PP* palmoplantar involvement; (+) hyperlinearity; (++) mild/moderate keratoderma; (+++) pronounced keratoderma. cDiagnosis based on histopathological/immunohistochemical/ultrastructural findings

**Table 4:** Clinicopathological findings of syndromic ichthyosis patient group

Target gene	Disease	Sample ID	Disease onset	Sex	Family history	Age at examination	Dermatological features	Extracutaneous findings	Histopathology/Immunopathology/Electron microscopy^d^
ALDH3A2	SLS	ID-32	5 mos	M	Yes ^a,b^	3 yrs	Diffuse scaling, sparing of the face	Psychomotor delay	NA
		ID-33	Birth	M	Yes^a,b^	10 yrs	Ichthyosiform erythroderma	Spastic paresis, psychomotor delay, failure to thrive	NA
		ID-34	Birth	F	Yes^c,b^	8 yrs	Generalized scaling	Spastic paresis, psychomotor delay	NA
MBTPS2	IFAP	ID-35	Birth	M	No^b^	1 yr	NA	NA	NA
PHYH	Refsum syndrome	ID-36	51 yrs	F	No^b^	66 yrs	Fine scaling	Ataxia and fine movement difficulties, sensorineural hypoacusia, retinitis pigmentosa, anosmia	NA
SNAP29-AP1S1	CEDNIK	ID-37	Birth	M	No	NA	NA	NA	NA
SPINK5	Netherton syndrome	ID-38	Birth	M	No	6 mos	Erythroderma, large thin scales, sparse hair	Bilateral conjunctivitis	Netherton syndrome
		ID-39	Birth	M	No	2 yrs	Erythroderma, large scales, fragile and brittle hair	Failure to thrive	Netherton syndrome
		ID-40	Birth	F	NA	12 yrs	NA	NA	NA
		ID-41	Birth	F	No	4 mos	Erythroderma and large scales	NA	NA
EBP	Conradi Hünermann Happle syndrome	ID-42	Birth	F	No	32 yrs	NA	Chondrodysplasia punctata	NA
		ID-43	Birth	F	No	3 mos	NA	Chondrodysplasia punctata	NA
VPS33B	ARC	ID-44	Birth	F	No^b^	5 yrs	Fine scaling	Arthrogryposis, elevated liver enzymes, plagio-brachycephaly, kneecap hypoplasia	NA
ABHD5	Neutral lipid storage disease	ID-45	Birth	M	Yes^a^	17 yrs	Erythroderma, fine whitish scales, reticulated skin on neck PP (++)., mild ectropion	Hepatic cirrhosis, myopathy, short stature	NA
		ID-46	Birth	F	Yes^c^	29 yrs	Erythroderma, small whitish scales, reticulated skin on joints, scales on the scalp, PP (+++), mild ectropion	Hepatic cirrhosis, myopathy	NA

^a^Sister; ^b^consanguineous parents, ^c^brother; *NA* not available, *Yr(s)* year(s), *mo(s)* month(s), *PP* palmoplantar involvement; (+) hyperlinearity; (++) mild/moderate keratoderma; (+++) pronounced keratoderma. ^d^Diagnosis based on histopathological/immunohistochemical/ultrastructural findings

*SLS* Sjögren-Larsson syndrome, *IFAP* Ichthyosis follicularis alopecia photophobia, *CEDNIK* Cerebral-dysgenesis-neuropathy-ichthyosis-palmoplantar keratoderma, *ARC* arthrogryposis-renal dysfunction-cholestasis syndrome

In the group of ARCI, 27 out of 31 patients presented genetic variants in different genes of the panel. Half of them carried a homozygous mutation, ten were compound heterozygotes while one patient had a hemizygous mutation and a microdeletion on the other allele. Involvement of *ALOX12B* was found in nine patients, *TGM1* in seven, *NIPAL4* in four, *ABCA12* in three, and *ALOXE3 and CYP4F22* in two patients each. The vast majority of mutations were missense (66.6 %), nonsense and splice site mutations were quite equally represented (12.9 % and 16.6 % respectively), while microdeletions and frameshift mutations were more rare (1.8 %) (Table 5). In the group of the X-linked ichthyosis thirteen individuals were analyzed for the *STS* gene: three had a missense mutation, eight had a genomic microdeletion and two were negative (Table 6). Keratinopathic ichthyosis was analyzed in five subjects: one had a heterozygous nonsense mutation in *KRT1*, in two a previously described missense mutation in *KRT10* was found, one patient had a codon deletion in *KRT2* while the last one was negative (Table 7).

**Table 5:** List of sequence variations detected in patients with ARCI and literature references

ARCI				
Target Gene	Sample ID	Variation 1	Variation 2	Note and references
NIPAL4	ID-1	c.527 C > A, p.A176D	c.527 C > A, p.A176D	[10]
	ID-2	c.527 C > A, p.A176D	c.527 C > A, p.A176D	[10]
	ID-3	c.527 C > A, p.A176D	c.527 C > A, p.A176D	[10]
	ID-4	c.527 C > A, p.A176D	deletion	[10]
ALOX12B	ID-5	c.1594 G > C, p.E532Q	c.130_131delGA, p.D44LfsX19	npd-npd
	ID-6	c.1002delG, p.Q334HfsX18	c.1579 G > A, p.V527M	npd - [11]
	ID-7	c.1412 T > C, p.L471P	c.1412 T > C, p.L471P	npd
	ID-8	c.1261 C > T, p.H421Y	c.1261 C > T, p.H421Y	[11]
	ID-9	c.71 T > C, p.L24P	c.1324 C > T, p.R442W	npd - npd
	ID-10	c.527 + 1G > A	c.340 C > T, p.R114W	npd - [12]
	ID-11	c.1384G > A,p.G462S	c.1384G > A,p.G462S	Npd
	ID-12	c.1192C > T,p.H398Y	c.1192C > T,p.H398Y	Npd
	ID-13	c.1192C > T,p.H398Y	c.1821 C > G, p.K607N	npd – npd
CYP4F22	ID-14	c.728 g > a. p.R243H	c.1084 C > T, p.R362X	[13] - npd
	ID-15	c.367 + 1G > A	c.1303C > T, p.H435Y	npd - [13]
TGM1	ID-16	c.814 T > C, p.S272P	c.877-2 A > G	[14, 15]
	ID-17	c.877-2 A > G	c.877-2 A > G	[15]
	ID-18	c.1187 G > A, p.R396H	c.919 C > G, p.R307G	[3–16]
	ID-19	c.919C > G,p.R307G	c.919C > G,p.R307G	[16]
	ID-20	c.788G > A,p.W263X	c.788G > A,p.W263X	[17]
	ID-21	c.401A > G,p.Y134C	c.401A > G,p.Y134C	[3]
	ID-22	c.1306C > G, p.H436D	c.1306C > G, p.H436D	npd - npd
ALOXE3	ID-23	c.1027C > T,p.R343X	c.2285C > T,p.P762L	npd - [12]
	ID-24	c.543 + 5delG	c.2461 C > T, p.R821W	npd – npd
ABCA12	ID-25	c.7105-2delA	c.7105-2delA	Npd
	ID-26	c.459T > G, p.Y153X	c.4579 + 5 G > A	npd – npd
	ID-27	c.4139 A > G, p.N1380S	c.6305dupA, p.Y2102X	[18] - npd
**/**	ID-28	neg		
**/**	ID-29	neg		
**/**	ID-30	neg.		
**/**	ID-31	neg		

*npd* not previously described, *neg* negative

**Table 6:** List of mutations detected in patients with X-linked ichthyosis

X-Linked Ichthyosis			
Target gene	Sample ID	Variation	Note
STS	ID-47	c.1075 G > A, p.G359R	Npd
STS	ID-48	c.1075 G > A, p.G359R	Npd
STS	ID-49	c.452 C > G, p.P151R	Npd
STS	ID-50	neg	
STS	ID-51	neg	
STS	ID-52	del	~1,5 Mb (6.552.712–8.115.153)
STS	ID-53	del	~1,6 Mb (6.489.877–8.131.810)
STS	ID-54	del	~1,5 Mb (6.552.712–8.097.511)
STS	ID-55	del	~1,6 Mb (6.489.877–8.131.810)
STS	ID-56	del	~1,6 Mb (6.467.006–8.131.810)
STS	ID-57	del	~1,5 Mb (6.552.712–8.078.155)
STS	ID-58	del	~1,5 Mb (6.552.712–8.097.511)
STS	ID-59	del	~1,5 Mb (6.552.712–8.097.511)

*npd* not previously described, *neg* negative, *del* deleted

The field “Note” contains the start and the stop points of the genomic rearrangements

**Table 7:** List of sequence variations detected in patients with keratinopathic ichthyosis and literature references

Keratinopathic ichthyosis			
Target gene	Sample ID	Variation	Note and references
KRT1	ID-60	c.1657_1677del, p.S557_G563del	[19]
KRT10	ID-61	c.466C > T, p.R156C	[20]
KRT10	ID-62	c.467G > A, p.R156H	[21]
KRT2	ID-63	c.561_563delCAA, p.N187del	Npd
KRT2	ID-64	neg	

*npd* not previously described, *neg* negative, *del* deleted

Syndromic ichthyoses include a broad group of heterogeneous rare diseases with different incidence and mutation rate. Among these we have collected patients suspected for Netherton syndrome, Sjögren-Larsson syndrome, Refsum syndrome, IFAP (ichthyosis follicularis-alopecia-photophobia)/Bresheck syndrome, Conradi-Hünermann-Happle syndrome, cerebral dysgenesis-neuropathy-ichthyosis-palmoplantar keratoderma syndrome (CEDNIK), mental retardation/enteropathy/deafness/peripheral neuropathy/ichthyosis/keratoderma syndrome (MEDNIK), neutral lipid storage disease and arthrogryposis-renal dysfunction-cholestasis (ARC) syndrome in most of whom we have identified a genetic defect (Table 8).

**Table 8:** List of sequence variations detected in patients with syndromic ichthyosis and literature references

Syndromic Ichthyosis					
Target gene	Disease	Sample ID	Variation 1	Variation 2	Note and references
ALDH3A2	SLS	ID-32	c.551 C > G, p.T184R	c.551 C > G, p.T184R	[22]
		ID-33	c.680delG, p.R227fsX3	c.1108_1110delCTC, p.L370del	npd - npd
		ID-34	c.680delG, p.R227fsX3	c.1108_1110delCTC, p.L370del	npd - npd
MBTPS2	IFAP	ID-35	c.1286 G > A, p.R429H	//	[23]
PHYH	Refsum syndrome	ID-36	c.683_684insG, p.G228fsX2	c.683_684insG, p.G228fsX2	[24]
SNAP29-AP1S1	CEDNIK	ID-37	neg		
SPINK5	Netherton syndrome	ID-38	c. 301 A > T, p.K101X	?	Npd
		ID-39	c.1732 C > T, p.R578X	c.1888–1 G > A	[25]
		ID-40	c.1431–12 G > A	c.1607 + 2 T > C	[26] - npd
		ID-41	neg		
EBP	CDPX2	ID-42	neg		
		ID-43	neg		
VPS33B	ARC	ID-44	neg		
ABHD5	Neutral lipid storage disease	ID-45	c.550 C > T, p.R184X	c.550 C > T, p.R184X	[27]
		ID-46	c.550 C > T, p.R184X	c.550 C > T, p.R184X	[27]

*npd* not previously described, *neg* negative, *del* deleted

*SLS* Sjögren-Larsson syndrome, *IFAP* Ichthyosis follicularis alopecia photophobia, *CEDNIK* Cerebral-dysgenesis-neuropathy-ichthyosis-palmoplantar keratoderma, *CDPX2* Conradi-Hünermann-Happle syndrome, *ARC* arthrogryposis-renal dysfunction-cholestasis syndrome

## Discussion

The molecular diagnosis of ichthyosis is a complex and articulate process that still represents an exciting challenge. Many resources and skills should be involved to obtain satisfactory results. High-throughput technologies can actually offer new opportunities in relation to the amount of genes potentially analyzed, the number of samples examined and the quality of results. NGS is an innovative technology that is able to massive-parallel sequence millions of DNA segments with high definition capability. It has a wide diffusion in many fields of biomedical research, but diagnostic applications for genetic diseases are still in progress. A previous study based on NGS in a cohort of 14 patients affected by ichthyosis has been published by Scott et al [8]. The authors underline that NGS can be used as an accurate and cost-effective tool in the molecular diagnosis of this group of diseases with marked reduction in costs.

We report our experience on a cohort of patients using a NGS approach on the Illumina MiSeq platform. The preparation of the genomic library using the TSCA Illumina kit was completed in two working days. Two days were spent to run the samples on the MiSeq. MiSeqReporter, AV and IGV2.3 software were used for result interpretation. All mutations identified by Miseq Reporter, were validated by Sanger sequencing using standard protocols. 16 genes of different size were analyzed for the non-syndromic ichthyosis for each patient in one experiment; the costs and the time of the analysis are significantly reduced. Moreover the possibility to analyze a large number of genes associated with various diseases allows to study also the cases with not well determined phenotypes, giving the opportunity to make new genotype-phenotype correlation. Using this innovative molecular approach we were able to find pathogenic mutations in 53 out of 64 patients resulting in a 82.8 % total detection rate. Moreover, considering the restricted, more frequent group of ARCI, 27 out 31 patients had a genetic diagnosis with an estimated detection rate of 87.1 %, corresponding to a higher sensitivity compared to currently reported data [2].

Among ARCI we observed an unexpected high number of 15 homozygous variants compared to the 18 compound heterozygotes. Segregation analysis of family members clarified that the significant frequency of homozygous patients was due to several consanguineous marriages and to some unrelated individuals belonging to restricted geographic areas in relationship to a founder effect. Both events seem to have a relevant role on the incidence of ichthyosis in Italy. Another interesting result from the analysis of the data is the high rate of novel sequence variations found compared to known mutations: about a half of them were never reported in literature and their pathogenic effect has been predicted from current data bases of variants and from the familial study indicating both parents as carriers.

**Fig. 1:**
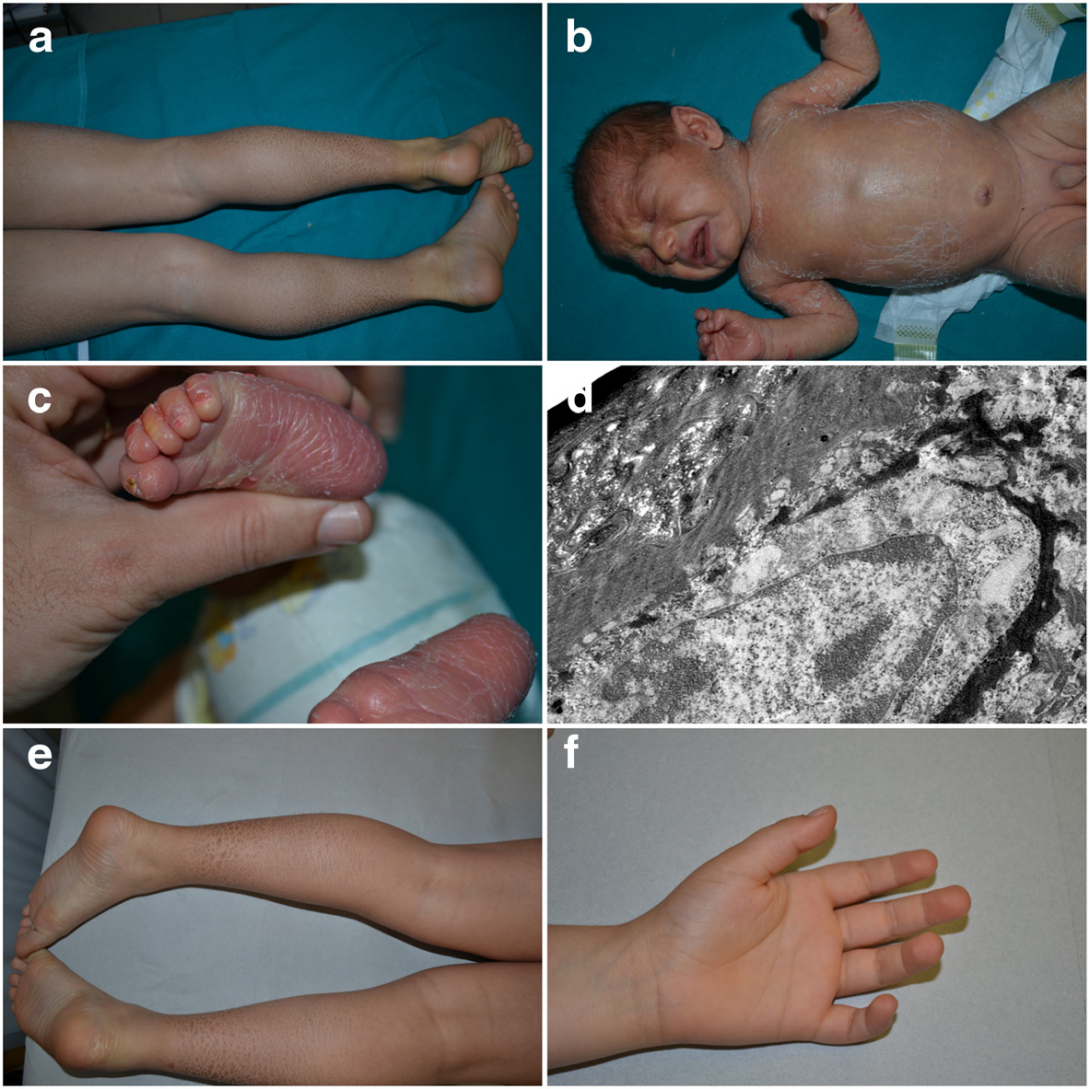
Clinical and ultrastructural findings in a subgroup of patients with X-linked ichthyosis. Patient ID-58 presented brown scales, sparing of antecubital and popliteal areas and normal palms and soles (**a**). Patient ID-52 showed residue of collodion membrane (**b**) and palmoplantar involvement (**c**). Electron microscopy of ID-52 displayed hyperkeratosis without lipid drops or membranous structures, but only rare residues of melanosomes ((**d**), Uranyl acetate-Lead citrate 3150x). Patient ID-49 presented brownish polygonal scales with sparing of folds (**e**), palms and soles (**f**)

In a subgroup of patients with X-linked ichthyosis discrepancies were found between clinical features and pathological observations. Patient ID-58 had been previously classified as vulgar ichthyosis on the basis of an orthokeratotic hyperkeratosis, absence of granular layer and reduced filaggrin staining. The child showed fine brown scales, sparing of antecubital and popliteal areas and normal palms and soles (Fig. 1a). Patient ID-52 was born as a collodion baby and had a brother who had been diagnosed with ichthyosis vulgaris. Once brought to our attention, the patient still had a residue of collodion membrane (Fig. 1b) and palmoplantar involvement (Fig. 1c). Therefore, after an uninformative histological and ultrastructural examination (Fig. 1d), he was placed in ARCI group according to the clinical history. In patient ID-53 anomalies of keratinosomes were highlighted in the ultrastructural study and lamellar ichthyosis was suspected. On physical examination ichthyotic brownish scales on legs and arms were observed together with sparing of folds, involvement of the scalp and absence of palmoplantar keratoderma.

Moreover, in patient ID-49 clinical history revealed maternal grandfather and two of his brothers affected by ichthyosis, giving evidence of an X-linked transmission, while electron microscopy suggested an ARCI. Physical examination showed brownish polygonal scales with sparing of folds (Fig. 1e), palms and soles (Fig. 1f).

In all these cases there were discrepancies between clinical features and histology or electron microscopy and only molecular analysis allowed to definitively resolve the diagnostic dilemma, demonstrating a deletion or mutation in *STS* (Table 6). In particular Patient ID-52, who was born as a collodion, shows that the age of disease onset can no longer be considered a diagnostic criterion, as already underlined by Oji V et al. [1].

**Fig. 2:**
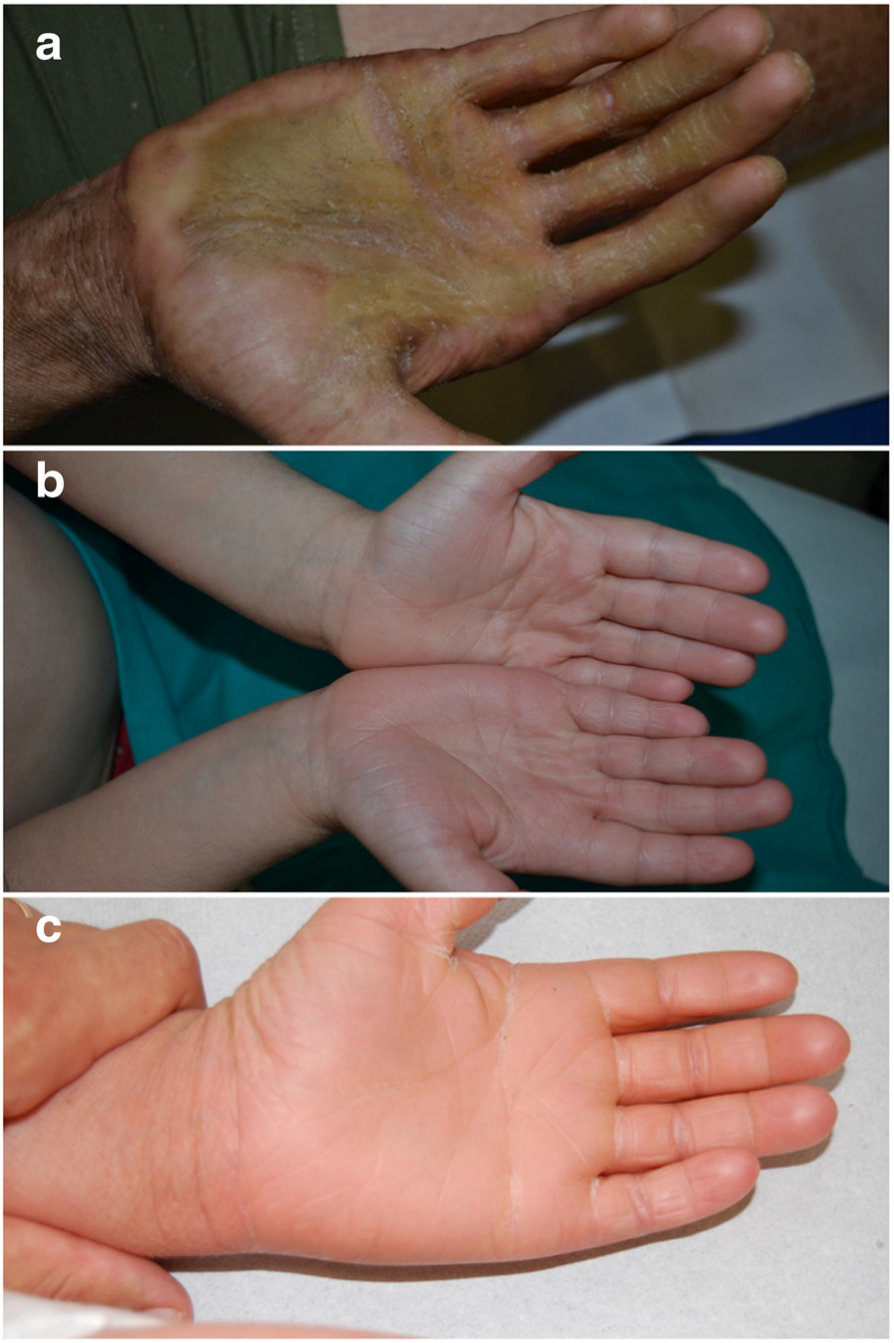
Palmar involvement in ARCI. Severe keratoderma in patient ID-4 with NIPAL4 mutation (**a**). Patient ID-24 with ALOXE3 mutation presented an ichthyosis vulgaris-like pattern (**b**). Patient ID-7 with ALOX12B mutation showed a moderate keratoderma (**c**)

Regarding the correlation between genotype and phenotype the appearance of the palmoplantar area is particularly interesting. As reported in literature, also in our patients ichthyosis vulgaris is characterized by accentuated markings, while X-linked usually is associated with sparing of palms and soles (Fig. 1f) [1, 9]. In the group of ARCI, *NIPAL4* mutation is associated with a pronounced keratoderma (Fig. 2a), while *ALOXE3* mutation is associated with an ichthyosis vulgaris-like pattern (Fig. 2b). *ALOX12B* and *CYP4F22* mutations usually produce moderate keratoderma (Fig. 2c).

The high number of patients enrolled in a relatively short time (about one year) is the result of the collaboration with the patient’s association and the Italian network of rare diseases that identifies the reference centers on the territory. Moreover, the activities on mutations analysis were also publicized through national conferences and publication on websites (www.ospedalebambinogesu.it, www.orpha.net). This organization has allowed the diagnosis of extremely rare and severe forms of ichthyosis. Therefore three patients affected by Sjögren-Larsson syndrome, one by Refsum syndrome, two by Netherton syndrome, one by IFAP/Bresheck syndrome and two by neutral lipid storage disease were diagnosed. A third case of Netherton syndrome had a partial molecular diagnosis because only a single pathogenic mutation was found. We investigated also the presence of haploinsufficiency but we were unable to demonstrate any genomic rearrangement. In this setting, the probability of a cryptic deep intronic variation affecting splicing, according to the clinical features, could be realistically taken into account.

Finally, the genetic diagnosis of ichthyosis leads to a more accurate and effective genetic counseling, allowing a correct evaluation of the risk of recurrence, particularly in families with consanguineous background. On this regard we have successfully managed several individuals, whose family members were affected from rare life-threatening diseases, such as harlequin ichthyosis, giving to the clinicians a needful tool to assess the reproductive risk.

## Conclusions

Our experience shows that the contribution to the diagnosis from anamnesis, physical examination, microscopy, and laboratory examination is variable. Therefore, a careful clinical selection of cases and a pathologic/clinical comparison are essential in order to guide the identification of the genetic mutation that today represents the diagnostic gold standard. In our experience this is underlined by the possibility of having inconsistencies between the clinical appearance and histopathology and by the detection in about half the cases of new genetic variants that are not described in the literature. Cases should be presented to the geneticist with a precise clinicopathological diagnosis and the multidisciplinary approach is the best method to rule out extracutaneous involvement and to improve the accuracy of the diagnostic process.

Nowadays is available a new innovative technology, NGS, that is more powerful, fast and economic and allows to more easily obtain genetic diagnosis.

### Ethics, consent and permissions

Blood samples were drawn from patients after obtaining informed consent.

### Consent to publish

We have obtained informed consent to publish individual data from the participants.

## Additional file

Additional file 1: Table S1. List of genes included in the diagnostic panel. (DOCX 85 kb)

## Abbreviations

NGS: Next-generation sequencing
ARCI: Autosomal recessive congenital ichthyosis
LI: Lamellar ichthyosis
CEI: Congenital ichthyosiform erythroderma
TSCA: TruSeq Custom Amplicon
DS: Design Studio
RTA: Real Time Analysis
GATK: Genome Analysis Toolkit
AV: Amplicon Viewer
IGV: Integrative Genome Viewer
aCGH: Array Comparative Genomic Hybridization
ARC: Arthrogryposis-renal-dysfunction-cholestasis
MEDNIK: Mental retardation-enteropathy-deafness-neuropathy-ichthyosis-keratoderma
CEDNIK: Cerebral dysgenesis-neuropathy-ichthyosis-palmoplantar keratoderma

## References

[1] Oji V, Tadini G, Akiyama M, Blanchet Bardon C, Bodemer C, Bourrat E (2010). Revised nomenclature and classification of inherited ichthyoses: results of the First Ichthyosis Consensus Conference in Sorèze 2009. J Am Acad Dermatol.

[2] Fischer J (2009). Autosomal recessive congenital ichthyosis. J Invest Dermatol.

[3] Farasat S, Wei MH, Herman M, Liewehr DJ, Steinberg SM, Bale SJ (2009). Novel transglutaminase-1 mutations and genotype-phenotype investigations of 104 patients with autosomal recessive congenital ichthyosis in the USA. J Med Genet.

[4] Liu L, Li Y, Li S, Hu N, He Y, Pong R (2012). Comparison of next-generation sequencing systems. J Biomed Biotechnol.

[5] Quail MA, Smith M, Coupland P, Otto TD, Harris SR, Connor TR (2012). A tale of three next generation sequencing platforms: comparison of Ion Torrent. Pacific Biosciences and Illumina MiSeq sequencers. BMC Genomics.

[6] Thorvaldsdóttir H, Robinson JT, Mesirov JP (2013). Integrative Genomics Viewer (IGV): high-performance genomics data visualization and exploration. Brief Bioinform.

[7] Robinson JT, Thorvaldsdóttir H, Winckler W, Guttman M, Lander ES, Getz G (2011). Integrative genomics viewer. Nat Biotechnol.

[8] Scott CA, Plagnol V, Nitoiu D, Bland PJ, Blaydon DC, Chronnell CM (2013). Targeted sequence capture and high-throughput sequencing in the molecular diagnosis of ichthyosis and other skin diseases. J Invest Dermatol.

[9] Traupe H, Fischer J, Oji V (2014). Nonsyndromic types of ichthyoses - an update. J Dtsch Dermatol Ges.

[10] Dahlqvist J, Klar J, Hausser I, Anton-Lamprecht I, Pigg MH, Gedde-Dahl T (2007). Congenital ichthyosis: mutations in ichthyin are associated with specific structural abnormalities in the granular layer of epidermis. J Med Genet.

[11] Lesueur F, Bouadjar B, Lefèvre C, Jobard F, Audebert S, Lakhdar H (2007). Novel mutations in ALOX12B in patients with autosomal recessive congenital ichthyosis and evidence for genetic heterogeneity on chromosome 17p13. J Invest Dermatol.

[12] Eckl KM, Krieg P, Küster W, Traupe H, André F, Wittstruck N (2005). Mutation spectrum and functional analysis of epidermis-type lipoxygenases in patients with autosomal recessive congenital ichthyosis. Hum Mutat.

[13] Lefèvre C, Bouadjar B, Ferrand V, Tadini G, Mégarbané A, Lathrop M (2006). Mutations in a new cytochrome P450 gene in lamellar ichthyosis type 3. Hum Mol Genet.

[14] Esposito G, Auricchio L, Rescigno G, Paparo F, Rinaldi M, Salvatore F (2001). Transglutaminase 1 gene mutations in Italian patients with autosomal recessive lamellar ichthyosis. J Invest Dermatol.

[15] Herman ML, Farasat S, Steinbach PJ, Wei MH, Toure O, Fleckman P (2009). Transglutaminase-1 gene mutations in autosomal recessive congenital ichthyosis: summary of mutations (including 23 novel) and modeling of TGase-1. Hum Mutat.

[16] Oji V, Hautier JM, Ahvazi B, Hausser I, Aufenvenne K, Walker T (2006). Bathing suit ichthyosis is caused by transglutaminase-1 deficiency: evidence for a temperature-sensitive phenotype. Hum Mol Genet.

[17] Hennies HC, Raghunath M, Wiebe V, Vogel M, Velten F, Traupe H (1998). Genetic and immunohistochemical detection of mutations inactivating the keratinocyte transglutaminase in patients with lamellar ichthyosis. Hum Genet.

[18] Lefévre C, Audebert S, Jobard F, Bouadjar B, Lakhdar H, Boughdene-Stambouli O (2003). Mutations in the transporter ABCA12 are associated with lamellar ichthyosis type 2. Hum Mol Genet.

[19] Richardson ES, Lee JB, Hyde PH, Richard G (2006). A novel mutation and large size polymorphism affecting the V2 domain of keratin 1 in an African-American family with severe, diffuse palmoplantar keratoderma of the ichthyosis hystrix Curth-Macklin type. J Invest Dermatol.

[20] Haruna K, Suga Y, Mizuno Y, Hasegawa T, Kourou K, Matsuba S (2007). R156C mutation of keratin 10 causes mild form of epidermolytic hyperkeratosis. J Dermatol.

[21] Mayuzumi N, Shigihara T, Ikeda S, Ogawa H (2000). Recurrent R156H mutation of KRT10 in a Japanese family with bullous congenital ichthyosiform erythroderma. J Eur Acad Dermatol Venereol.

[22] Rizzo WB, Carney G, Lin Z (1999). The molecular basis of Sjögren-Larsson syndrome: mutation analysis of the fatty aldehyde dehydrogenase gene. Am J Hum Genet.

[23] Oeffner F, Fischer G, Happle R, König A, Betz RC, Bornholdt D (2009). IFAP syndrome is caused by deficiency in MBTPS2, an intramembrane zinc metalloprotease essential for cholesterol homeostasis and ER stress response. Am J Hum Genet.

[24] Jansen GA, Waterham HR, Wanders RJ (2004). Molecular basis of Refsum disease: sequence variations in phytanoyl-CoA hydroxylase (PHYH) and the PTS2 receptor (PEX7). Hum Mutat.

[25] Sprecher E, Chavanas S, DiGiovanna JJ, Amin S, Nielsen K, Prendiville JS (2001). The spectrum of pathogenic mutations in SPINK5 in 19 families with Netherton syndrome: implications for mutation detection and first case of prenatal diagnosis. J Invest Dermatol.

[26] Capri Y, Vanlieferinghen P, Boeuf B, Dechelotte P, Hovnanian A, Lecomte B (2011). A lethal variant of Netherton syndrome in a large inbred family. Arch Pediatr.

[27] Ml A, Sawamura D, Nomura Y, Sugawara M, Shimizu H (2003). Truncation of CGI-58 protein causes malformation of lamellar granules resulting in ichthyosis in Dorfman-Chanarin syndrome. J Invest Dermatol.

