# Supplementary material for: Role of molecular testing in the multidisciplinary diagnostic approach of ichthyosis
Source: Orphanet J Rare Dis. 2016 Jan 13;11:4. doi: 10.1186/s13023-016-0384-4 (PMC4712481; doi:10.1186/s13023-016-0384-4)
Supplement: Additional file 1: Table S1. — List of genes included in the diagnostic panel. (DOCX 85 kb) [file 13023_2016_384_MOESM1_ESM.docx]

**Additional file 1: Table S1 List of genes included in the diagnostic panel**

| **Non-Syndromic Ichthyosis** | |
| --- | --- |
| ***Gene*** | ***Disease*** |
| *ABCA12* | ARCI, Autosomal Recessive Congenital Ichthyosis |
| *ALOX12B* | ARCI, Autosomal Recessive Congenital Ichthyosis |
| *ALOXE3* | ARCI, Autosomal Recessive Congenital Ichthyosis |
| *CYP4F22* | ARCI, Autosomal Recessive Congenital Ichthyosis |
| *NIPAL4* | ARCI, Autosomal Recessive Congenital Ichthyosis |
| *PNPLA1* | ARCI, Autosomal Recessive Congenital Ichthyosis |
| *TGM1* | ARCI, Autosomal Recessive Congenital Ichthyosis |
| *LIPN* | ARCI, Autosomal Recessive Congenital Ichthyosis |
| *GJB3* | Erythrokeratodermia Variabilis |
| *GJB4* | Erythrokeratodermia Variabilis |
| *POMP* | Keratosis Linearis with Ichthyosis Congenita and Sclerosing Keratoderma (KLICK) |
| *LOR* | Loricrin Keratoderma |
| *STS* | X-linked Ichthyosis |
| *KRT1* | Epidermolytic Ichthyosis |
| *KRT2* | Superficial Epidermolytic Ichthyosis |
| *KRT10* | Epidermolytic Ichthyosis |
| **Syndromic Ichthyosis** | |
| ***Gene*** | ***Disease*** |
| *VPS33B* | Arthrogryposis-Renal Dysfunction-Cholestasis (ARC) |
| *ALDH3A2* | Sjogren–Larsson Syndrome |
| *ABHD5* | Neutral Lipid Storage Disease |
| *SPINK5* | Netherton Syndrome |
| *GJB2* | Keratitis Ichthyosis Deafness (KID) |
| *EBP* | Conradi–Hünermann–Happle Syndrome |
| *MBTPS2* | Ichthyosis Follicularis-Alopecia-Photophobia (IFAP) |
| *SUMF1* | Multiple Sulfatase Deficiency |
| *NSDHL* | Congenital Hemidysplasia with Ichthyosiform Nevus and Limbs Defects Syndrome (CHILD) |
| *SNAP29* | Cerebral Dysgenesis, Neuropathy, Ichthyosis, Palmoplantar Keratoderma Syndrome (CEDNIK) |
| *AP1S1* | Mental Retardation, Enteropathy, Deafness, Peripheral Neuropathy, Ichthyosis, Keratodermia Syndrome (MEDNIK) |
| *SLC27A4 (FATP4)* | Ichthyosis Prematurity Syndrome |
| *PHYH* | Refsum Syndrome |
| *PEX7* | Refsum Syndrome |
| *GBA* | Gaucher Disease Type 2 |
| *ST14* | Ichthyosis Hypotrichosis Syndrome |
| *CLDN1* | Ichthyosis, Hypotrichosis, Sclerosing Cholangitis |
| *C7ORF11* | Trichothiodystrophy |
| *GTF2H5* | Trichothiodystrophy |
| *ERCC2* | Trichothiodystrophy |
| *ERCC3* | Trichothiodystrophy |
